# Migration of mitochondrial DNA in the nuclear genome of colorectal adenocarcinoma

**DOI:** 10.1186/s13073-017-0420-6

**Published:** 2017-03-29

**Authors:** Vinodh Srinivasainagendra, Michael W. Sandel, Bhupendra Singh, Aishwarya Sundaresan, Ved P. Mooga, Prachi Bajpai, Hemant K. Tiwari, Keshav K. Singh

**Affiliations:** 1 Department of Biostatistics, School of Public Health, University of Alabama at Birmingham, Birmingham, Alabama 35294 USA; 2 Department of Genetics, University of Alabama at Birmingham, Birmingham, Alabama 35294 USA; 3 Departments of Genetics, Environmental Health, Center for Free Radical Biology, Center for Aging and UAB Comprehensive Cancer Center, University of Alabama at Birmingham, Birmingham, Alabama 35294 USA; 4 Departments of Pathology, Environmental Health, Center for Free Radical Biology, Center for Aging and UAB Comprehensive Cancer Center, University of Alabama at Birmingham, Birmingham, Alabama 35294 USA; 5 Birmingham Veterans Affairs Medical Center, Birmingham, Alabama 35294 USA; 6 Department of Genetics, School of Medicine, University of Alabama at Birmingham, Kaul Genetics Building, Suite 620, 720 20th St. South, Birmingham, AL 35294 USA; 7 Present address: Department of Biological and Environmental Sciences, School of Natural Sciences and Mathematics, University of West Alabama, Livingston, Alabama USA

**Keywords:** Cancer, Tumor, Colorectal cancer, Mitochondria, Mitochondrial DNA, YME1L1, NUMT, Numtogenesis, mtDNA transfer, Genetic instability

## Abstract

**Background:**

Colorectal adenocarcinomas are characterized by abnormal mitochondrial DNA (mtDNA) copy number and genomic instability, but a molecular interaction between mitochondrial and nuclear genome remains unknown. Here we report the discovery of increased copies of nuclear mtDNA (NUMT) in colorectal adenocarcinomas, which supports link between mtDNA and genomic instability in the nucleus. We name this phenomenon of *nu*clear occurrence of *m*i*t*ochondrial component as numtogenesis. We provide a description of NUMT abundance and distribution in tumor versus matched blood-derived normal genomes.

**Methods:**

Whole-genome sequence data were obtained for colon adenocarcinoma and rectum adenocarcinoma patients participating in The Cancer Genome Atlas, via the Cancer Genomics Hub, using the GeneTorrent file acquisition tool. Data were analyzed to determine NUMT proportion and distribution on a genome-wide scale. A NUMT suppressor gene was identified by comparing numtogenesis in other organisms.

**Results:**

Our study reveals that colorectal adenocarcinoma genomes, on average, contains up to 4.2-fold more somatic NUMTs than matched normal genomes. Women colorectal tumors contained more NUMT than men. NUMT abundance in tumor predicted parallel abundance in blood. NUMT abundance positively correlated with GC content and gene density. Increased numtogenesis was observed with higher mortality. We identified *YME1L1*, a human homolog of yeast *YME1* (yeast mitochondrial DNA escape 1) to be frequently mutated in colorectal tumors. *YME1L1* was also mutated in tumors derived from other tissues. We show that inactivation of *YME1L1* results in increased transfer of mtDNA in the nuclear genome.

**Conclusions:**

Our study demonstrates increased somatic transfer of mtDNA in colorectal tumors. Our study also reveals sex-based differences in frequency of NUMT occurrence and that NUMT in blood reflects NUMT in tumors, suggesting NUMT may be used as a biomarker for tumorigenesis. We identify *YME1L1* as the first NUMT suppressor gene in human and demonstrate that inactivation of *YME1L1* induces migration of mtDNA to the nuclear genome. Our study reveals that numtogenesis plays an important role in the development of cancer.

**Electronic supplementary material:**

The online version of this article (doi:10.1186/s13073-017-0420-6) contains supplementary material, which is available to authorized users.

## Background

Natural transfer of mitochondrial DNA (mtDNA) into the nuclear genomes of eukaryotic cells is a well-established and evolutionarily ongoing process. The nuclear copies of mtDNA are described as NUMTs (nuclear mtDNA sequences) [1]. Frequently, intact mitochondria containing mtDNA, mitochondrial RNA (mtRNA), and mitochondrial proteins are also reported to localize into the nucleus [2–9]. We have named this phenomenon of occurrence of *nu*clear *m*i*t*ochondria as *numtogenesis.* We define numtogenesis as the occurrence of any mitochondrial components into the nucleus or nuclear genome. Numtogenesis is reported in at least 85 sequenced eukaryotic genomes [1]. These include human, plant, yeast, fruit fly, *Plasmodium*, *Caenorhabditis*, and other species [1, 10, 11]. In human, NUMT insertions are estimated to occur at a rate of ~5 × 10^−6^ per germ cell per generation [12].

Evolutionary studies suggest that the origin and insertion of germline NUMTs are distributed non-randomly in humans and other mammals [13–15]. Germline NUMTs tend not to originate from the mtDNA displacement loop (“d-loop”), and they tend to be located in damage-prone regions of the nuclear genome, such as open chromatin and fragile sites [13–15]. These studies implicate NUMTs in double-strand break repair [13]. The mechanism(s) of NUMT accumulation is not well understood. It is suggested that mitochondria migrate towards the nucleus and accumulate near the nuclear membrane [16–18]. The most parsimonious mechanism explaining NUMT accumulation involves de novo transposition from the mitochondrion to the nucleus; however, NUMTs are also known to accumulate via segmental duplication (sometimes within repetitive elements), and possibly RNA retro-transposition [19–21]. The human genome contains between 755 and 1105 germline NUMTs, with mtDNA identities ranging from 64–100% [12, 22]. Germline NUMTs with the lowest similarity to mtDNA have been evolutionarily conserved for tens of millions of years, while the most recent insertions occurred after certain *Homo sapiens* populations migrated to Eurasia [22–26]. Human germline NUMTs are relatively well described but little is known about the somatic NUMT and its role in human pathology [27].

We and others have demonstrated that mito-nuclear interactions play a key role in tumorigenesis [28–35], but a role of mtDNA integration within the nuclear genome remains relatively unexplored. In this study, we analyzed the prevalence of NUMT in colorectal cancer (CRC) because a number of mitochondrial associations are relatively well characterized in CRC. There is a reported relationship between CRC risk and mtDNA copy number [36–38], and there are associations between germline mtDNA variants and CRC risk and mortality [39, 40]. Similarly, colorectal adenocarcinomas tend to have aberrant mtDNA copy number and somatic variant frequencies compared to matched blood-derived normal genomes [41–44]. We differentiated two classes of NUMTs with distinct characteristics, those that are inherited in the germline and those that are somatic NUMTs acquired during tumorigenesis. We present the first quantitative analysis on the abundance of somatic NUMTs in human colorectal adenocarcinoma genomes relative to matched blood-derived normal samples. Further, we compare the distributions of somatic NUMTs and germline NUMTs, describe sex-based differences in numtogenesis, and demonstrate that NUMT abundance in blood reflects NUMT abundance in tumor. In addition, we identify *YME1L1* as the first “NUMT suppressor” gene in humans whose inactivation leads to increased numtogenesis.

## Methods

### Data harvesting

Whole-genome sequence data were obtained for colon adenocarcinoma (COAD) and rectum adenocarcinoma (READ) patients participating in The Cancer Genome Atlas (TCGA), via the Cancer Genomics Hub (CGHub) [45], using the GeneTorrent file acquisition tool. In order to appraise alternative protocols for transactions on big-data endpoints, we evaluated GTFuse, an innovate software that offers faster access to DNA sequence data without the need for staging the data locally. Although data bandwidth hungry, GTFuse still enabled us to rapidly prototype our research on selected regions of the genome, thereby indicating signals of NUMT deposition in the nuclear genome. A symbiotic combination of GeneTorrent and GTFuse was used to choreograph the downstream data analysis pipeline. Our data harvester robots adopt a high-throughput analysis model by spreading GTFuse across a computational cluster fabric available locally on-campus At the time of manuscript generation, although the GTFuse web-link within AnnaiSystems webportal was unavailable, an alternate web URL https://annaisystems.zendesk.com/hc/en-us was found through web search means.

### Quality control

TCGA sequence data for individuals with matched colorectal tumor and blood-derived normal samples were harvested from CGHub. Downloaded data went through an intense quality control (QC) pipeline, which involved the use of (a) clinical information downloaded from TCGA Data Matrix, (b) short-reads alignment statistics derived from the Binary AlignMent (BAM) sequence data downloaded from CGHub, and (c) tagging and elimination of duplicate reads in the alignment data (BAM) using a popular next-generation sequencing tool, Picard 2.5. All sequence data used in this study were generated at Harvard Medical School using Illumina paired-end read technologies GAII and HiSeq, and reads were mapped to the hg18 human reference assembly. Since paired-end read technology offers better mapping coverage, improved directional sequence accuracy, and reliable mapping of reads to a reference genome, we used sequencing datasets generated using Illumina’s paired-end read technology as opposed to single-end read technology. Paired-end reading, with its increased coverage across several genomic bases, also improves the ability to identify relative positions of the two ends of a single read, especially when each read-end maps to different genomes, nuclear and mitochondrial.

**Fig. 1:**
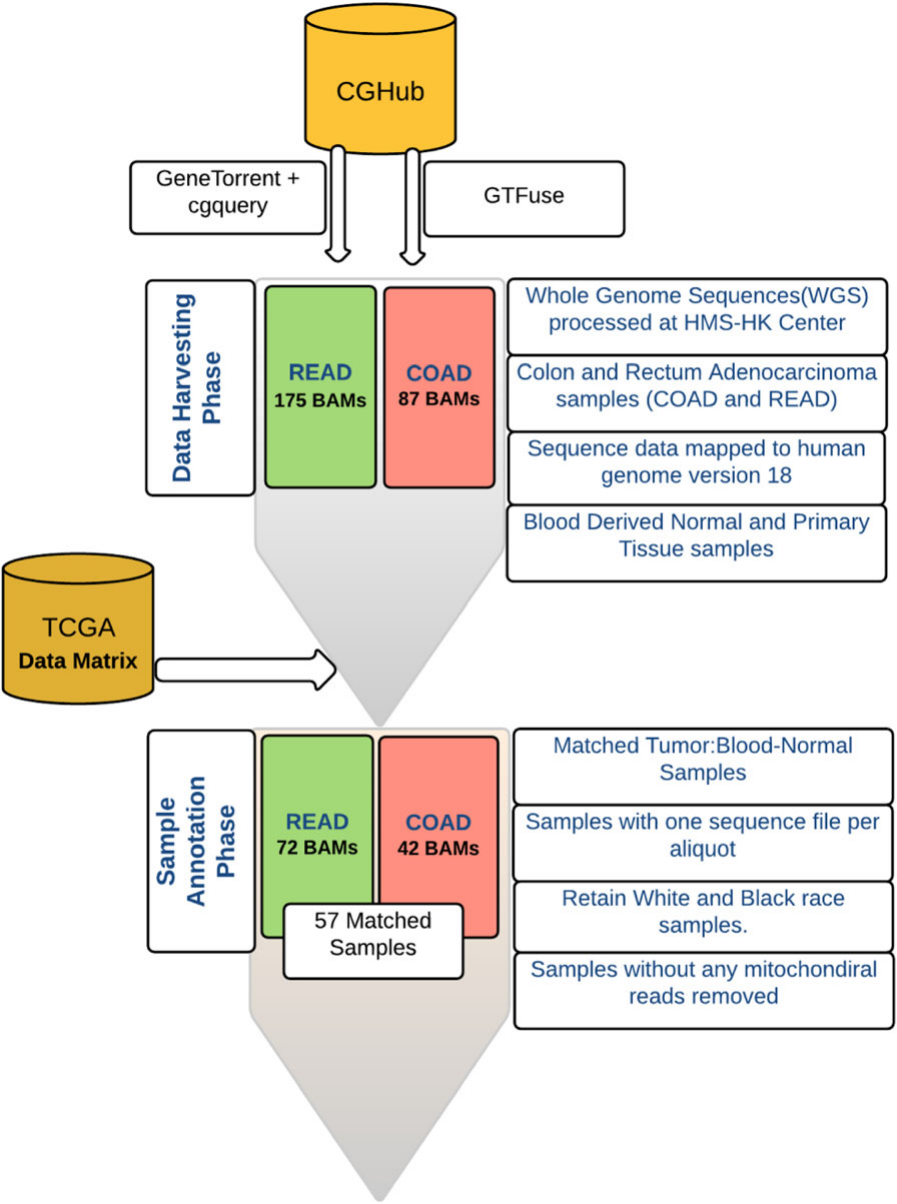
Quality control (QC) pipeline. Upstream QC pipeline conducted on aligned sequence data processed at Harvard Medical School (HMS-HK) was downloaded from TCGA CGHub. Clinical annotations for the matched tumor and blood-derived normal samples were downloaded from TCGA data matrix. COAD colon adenocarcinoma, READ rectum adenocarcinoma, HMS-HK Harvard Medical School

Further, a second-level QC was conducted based on the clinical annotations of the samples (Fig. 1). Participants were excluded a priori if even one of the following conditions as unmet (a) one or more elements of TCGA barcode differed between matched tumor and blood-derived healthy samples (i.e., sample, vial, analyte, and plate identifiers), (b) multiple alignment data (BAM files) per aliquot were reported when using CGHub’s sequence data query tool, cgquery, and (c) self-reported race and ethnicity status was not white or black. After the complete QC process, the final dataset included 57 colorectal tumor and 57 matched blood-derived normal genome alignment files (BAM files). Figure 1 illustrates the complete QC pipeline.

### Data analytics

A two-pronged analytic approach is taken to determine (a) NUMT proportion and distribution on a genome-wide scale and (b) hot spots in the nuclear and mitochondrial genomes experiencing more than blood-derived healthy (normal) NUMT abundance.

The primary obstacle to quantifying non-homologous recombinant elements from high-throughput sequence data is the reliable detection of reads that map to the sequence breakpoint. Although conceptually intuitive, the mapping and alignment of breakpoint reads is complicated by the large number of NUMT inserts that are not represented in the reference genome sequence. Alternatively, de novo genome assembly is computationally intensive, and any low-stringency mapping algorithm that would accurately identify insertion sites would also return many false positive hits. Fortunately, paired-end read technology offers a convenient and reliable method of detecting genome structural rearrangements, effectively bypassing an intensive search for non-homologous breakpoints. In the mapping step, paired-end coordinates are recorded for each read, which we filtered using simple text edit scripts. We used utilities of the SAMtools framework to quantify all paired-end reads that mapped to different chromosomes (mismatches). Further NUMTs were filtered from the mismatch output files, where one paired end mapped to mtDNA and the other to a nuclear coordinate. Since breakpoints were not precisely identified, we used the paired-end coordinate as a proxy for nuclear insertion site because the average distance between properly mapped reads was short (ca*.* 150 bp).

In addition to challenges presented by NUMT detection, we recognized challenges related to data normalization that are not addressed by traditional copy-number analyses. NUMTs represent a subset of the mapped genomic read count, and local representation cannot be assumed constant across tumor genomes. To study the NUMT abundance, we defined a metric for calculating relative NUMT abundance from high-throughput genomic sequence data. First, relative NUMT abundance was normalized to mapped read count across a given genomic region, expressed as a ratio (*P*_*ij*_):

$$ {P}_{ij}=\frac{N_{ij}}{M_{ij}} $$

Let *N*_*ij*_ and *M*_*ij*_ represent NUMT read count and mapped read count within a genomic interval (*i*), respectively, for genome type (*j*) for an individual; where a genomic interval is defined as whole genome (*gen*), chromosome (*chr*), chromosome arm (*arm*), or 2.5-mbp sliding window (*win*); and where genome type (*j*) is either tumor (*t*) or matched blood-derived healthy (*h*) genome of an individual. The change in tumor and matched healthy NUMT proportions is defined as *ΔP*_*i*_ = *P*_*it*_ − *P*_*ih*_ and the ratio is given as:

$$ {R}_i={P}_{i t}\ast {\left({P}_{i h}\right)}^{-1} $$

where *R*_*i*_ represents the proportional fold-change in NUMT abundance within a genomic region (*i*) for tumor (*t*) versus matched blood-derived healthy (*h*) genomes. When sub-genomic regions (e.g., sliding windows and cytobands) were compared, *P*_*ih*_ was replaced with ${\overline{P}}_{ih}$, where ${\overline{P}}_{ih}$ is the average NUMT proportion within the genomic interval for all blood-derived healthy samples sharing the same plate barcode to avoid zero denominator because blood-derived healthy samples may not have any NUMTs within the short regions.

The *R*_*i*_ derived earlier can be less than or greater than 1 due to the asymmetric property of ratios (e.g., where *R*_*i*_ = 0.5 represents a change in the opposite direction, i.e., there are twice as many NUMTs in healthy samples compared to tumor; and similarly, R_i_ = 2.0 represents a change in the positive direction, i.e., there are twice as many NUMTs in tumor samples compared to their matched blood-derived normal samples). In order to compare NUMTs between tumor and blood-derived healthy samples within the genomic regions, it is necessary to rescale *R*_*i*_ values less than one and to collapse values of one and negative one to zero. Further, we defined an indicator value *S*_*i*_ to denote the direction of change between tumor and matched blood-derived normal NUMT abundance:

$$ {S}_i=\frac{\varDelta {P}_i}{\left|\varDelta {P}_i\right|} $$

and:

$$ {R}_i^{/} = {R}_i^{S_i}\ast {S}_i $$

Let the *R*_*i*_^/^represent the rescaled proportional fold-change in relative NUMT abundance between tumor and matched blood-derived normal samples. *R*_*i*_^/^was calculated for the nuclear genome using five nested data partitions, where *i* represents whole genomes (*gen*), chromosomes (*chr*), chromosome arms (*arm*), cytobands (*cyt*), and sliding windows (*win*). For nuclear genome-scale comparisons, normalization to mapped reads (*M*_*ij*_) did not include sex chromosomes due to sequence representation bias associated with differences in chromosome length. For small-partition comparisons (e.g., sub-band), samples were pooled before normalization to avoid bias associated with zero denominators. For the mitochondrial genome, *R*_*i*_^/^was calculated for three nested data partitions, where *i* represents genome (*mtg*), replication strand (*mst*), and gene (*mgn*).

The relationship between *R*_*arm*_^/^and mapped read count was evaluated with linear regression to assess whether NUMT transposition is coincident with aneuploidy. The relationships between *R*_*win*_^/^were evaluated between GC content to assess whether transcriptional activity predisposes the nuclear genome to integration of non-homologous DNA. All statistical analyses were conducted in the R statistical environment (http://www.R-project.org/)*.* The NUMT proportions and abundance in blood-derived normal and primary tumor sites are quantified in Additional file 1: Table S1.

### *YME1L1* gene knockout and NUMT analyses in isolated nuclear DNA

Using the CRISPR-Cas9 method, we knocked out the human *YME1L1* gene in human breast epithelial MCF-7 cells as described earlier [46]. To avoid any mtDNA contamination in NUMT analyses, we isolated nuclear fractions free of mitochondrial contamination. Briefly, *YME1L1* knockout and wild-type MCF-7 cells were lysed using lysis buffer (10 mM HEPES, pH 7.9, 10 mM KCl, 0.1 mM EDTA) containing 10% IGEPAL detergent for 10 min at room temperature and centrifugation at 15,000xg for 3 minutes was carried out to pellet the intact nuclei. To completely avoid cytoplasmic fraction contamination in the nuclear pellet, the lysis step was repeated one more time. The purity of the nuclear fraction was ascertained by performing western blotting for mitochondrial encoded cytchrome oxidase II (COXII) protein. Nuclear DNA was prepared from the nuclear pellet using the NAOH boiling method [47]. Mitochondrial DNA content in the nuclear fraction was analyzed by real-time PCR by absolute quantification using primers for *COXII* (mtDNA-encoded gene) and *Beta-2 microglobulin* (*B2M*; nuclear DNA-encoded gene). *B2M* served as an internal control.

### Yeast transformation and genetic selection

Yeast expression vectors (pYX113, pPT31-yYme1, and pYX113-hYme1L) were transformed using the lithium acetate, single-stranded DNA, polyethylene glycol method [48–50]. Following transformation, yeast harboring the desired vector was selected using synthetic drop-out (SD) medium (0.67% [w/v] nitrogen base without amino acids, 0.07% [v/v] drop-out amino acid mix (-His/-Trp/-Ura), 0.02% [w/v] L-histidine and excluding the amino acid that is a selectable marker, 2% [w/v] dextrose, and 1.5% agar for agar plates). Single cell colonies from plates lacking uracil were cultured in glucose medium and 1 × 10^4^ and 5 × 10^7^ or 5 × 10^8^ cells were plated in triplicate onto YPD and SD media lacking tryptophan.

The yeast strains throughout this study were grown in YPD medium (1% [w/v] yeast extract, 2% [w/v] bactopeptone, 2% [w/v] dextrose) or YPG medium (1% [w/v] yeast extract, 2% [w/v] bacto-peptone, 3% [w/v] glycerol, pH 4.9) at 30 °C. The yeast strains constructed and used in this study are detailed in Table 1. The yeast Yme1-1 strain (PTY62) was transformed with a plasmid expressing the yeast *YME1* (yYme1) or human *YME1L1* (hYme1L1) gene under the alcohol dehydrogenase (ADH) promoter.

**Table 1:** *Sacharomyces cerevisiae* strains used in the study

Strain	Genotype	Mitochondrial genotype
PTY62 *Yme1-1*	*MATa ura3-52 lys2 leu2-3, 112 trp1-Δ1 yme1-1*	*ρ + (TRP1)*
PTY62- yYme1		
PTY62-hYme1L1		

## Results

### Adenocarcinoma genomes contain increased NUMTs compared to healthy genomes

**Fig. 2:**
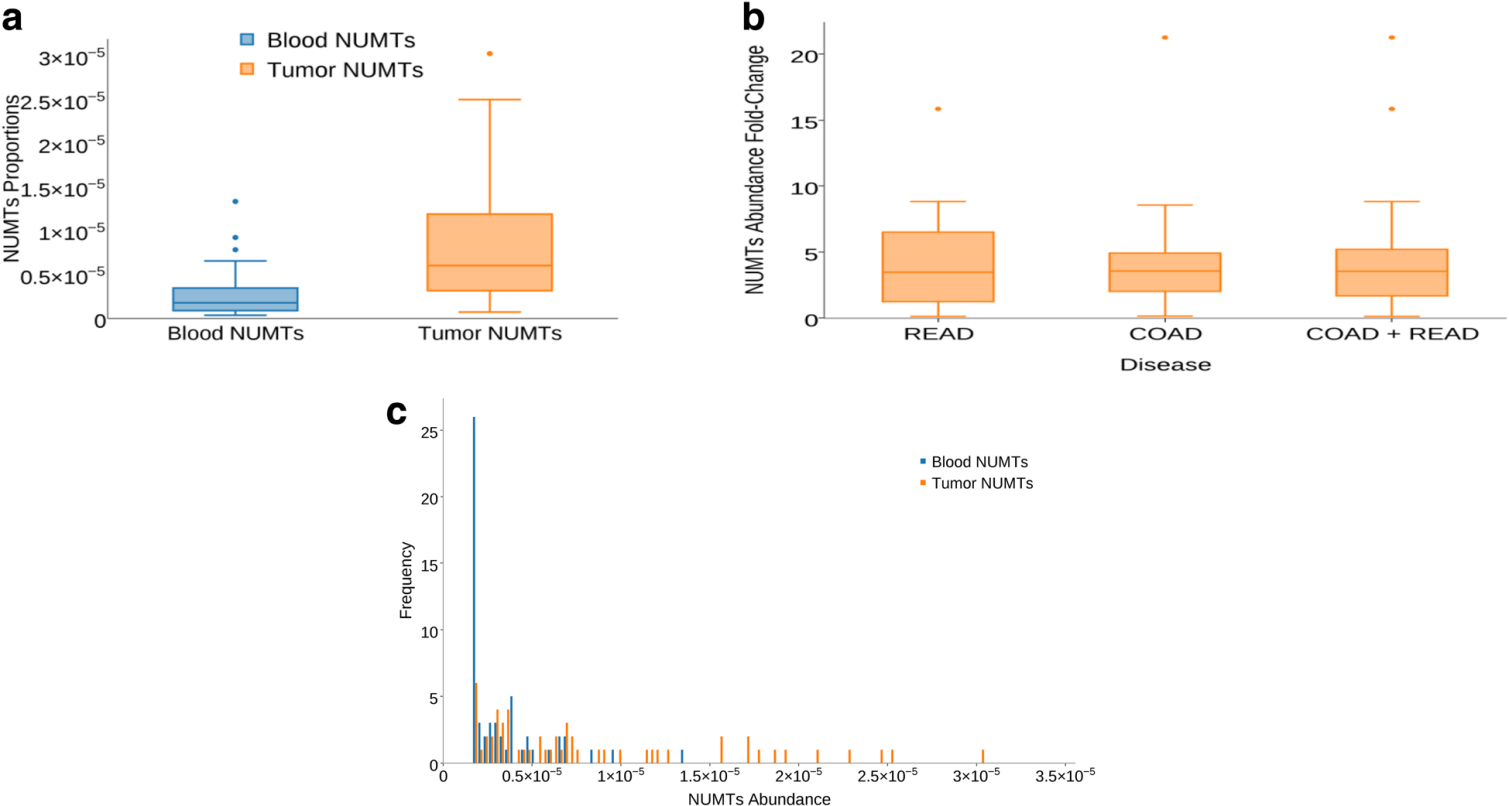
Distribution of NUMT proportions in tumor and normal genomes. **a** Distribution of NUMT proportions in 57 samples with matched tumor and blood-derived normal genomes. NUMT proportion is defined as the ratio between NUMT read count and total mapped read count on a genome-wide scale. The mean and standard deviation, respectively, across the 57 samples are 8.31 × 10^−6^ and 7.11 × 10^−6^ for tumor genomes and 2.65 × 10^−6^ and 2.49 × 10^−6^ for normal genomes. A two-tailed paired *t*-test conducted between the NUMT proportions for 57 (COAD + READ) samples revealed a *P* value of 1.63 × 10^−5^. When comparing the NUMT proportions between the cancer site group versus blood-derived group using a two-tailed unequal variance *t*-test, a *P* value of 1.43 × 10^−5^ was observed for COAD samples (*N* = 36) and 3.82 × 10^−3^ for READ samples (*N* = 21). **b** Fold change in the tumor NUMT proportions compared to blood-derived normal genomes across colon (*COAD*) and rectum (*READ*) cancer samples. Tumor genomes contained 4.42-fold more NUMTs than blood-derived normal genomes. A two-tailed unequal variance *t*-test conducted between the NUMT abundance of colon cancer samples (*N* = 36) and rectal cancer samples (*N* = 21) revealed a *P* value of 0.91, indicating no difference in the NUMT abundance between the two cancer sites, colon and rectum. **c** Right-skewed distribution, log transformed, and one-tailed paired *t*-test performed on tumor and matched blood-derived normal samples to determine the statistical significance (*P* value 8.79 × 10^−13^) of the log-transformed NUMT abundance levels

Since mitochondrial abnormalities are frequently described in cancer, we asked whether mitochondrial dysfunction results in increased prevalence of NUMTs in tumors. Constitutively, tumor genomes contained more NUMTs than matched blood-derived healthy genomes when normalized to mapped read pairs. When scanned for NUMT proportion and distribution on a genome-wide scale across 57 samples, tumor genomes contained, on average, 4.42-fold more NUMTs than healthy normal genomes. Box plots (Fig. 2a, b) indicate the mean, dispersion, and skewness of NUMT density for the genome groups. Although both tumor and matched normal genomes show right-skewed distributions (Fig. 2c), the third quartile (3.43 × 10^−6^) of the normal blood genome group is less than the first quartile (3.13E-6) of the tumor genomes, indicating significant difference in the NUMT density. A one-tailed, paired *t*-test was performed on tumor and matched blood-derived healthy samples to determine the statistical significance (*P* value 8.79 × 10^−13^) of the log transformed NUMT abundance levels. In one case, however, 22-fold more tumor NUMTs were observed in a Caucasian woman. This individual’s vital status was pronounced as deceased after reporting her pathologic tumor stage as I. These studies suggest that colorectal adenocarcinomas contain increased NUMTs compared to blood-derived healthy NUMTs.

### NUMT abundance in blood correlates with NUMT abundance in tumor

**Fig. 3:**
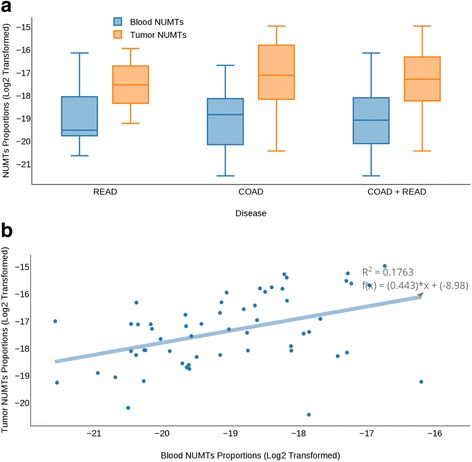
Log2-transformed NUMT proportions in tumor and blood-derived normal (Pij) genomes. **a** Log2-transformed NUMT proportions in tumor and normal genomes showcasing the difference in their means, indicating higher NUMT distribution in tumor genomes compared to matched blood-derived normal genomes. A two-tailed paired *t*-test conducted between the log2-transformed NUMT proportions for 57 samples revealed a *P* value of 1.87 × 10^−11^, showing significant difference in the log-transformed NUMT proportions between primary tumors and blood-derived normal genomes. **b** Relationship between abundance measures of blood-derived normal genome NUMTs and tumor genome NUMTs showing a positive relationship with R^2^ = 0.17 and *P* value = 0.0016

Increasingly, molecular characteristics of tumors are demonstrated to be reflected in the blood of cancer patients. We therefore envision that increased NUMT incidence observed in tumors may be found in the matched blood of cancer patients. In order to determine the predictability of tumor NUMT density based on the NUMT frequency in matched blood-derived healthy samples, a simple linear regression was performed on the log base 2-transformed proportions of NUMTs in blood-derived healthy genomes (Pgenh) and tumor genomes (Pgent). Linear regression revealed a positive relationship between Pgent and Pgenh with an R^2^ = 0.17, *P* value = 0.0016 (Fig. 3a, b). These results indicate that there is a positive correlation between proportions of NUMTs in blood-derived and primary tumor in colorectal cancer samples.

### Colorectal tumors in women harbor more NUMTs

**Fig. 4:**
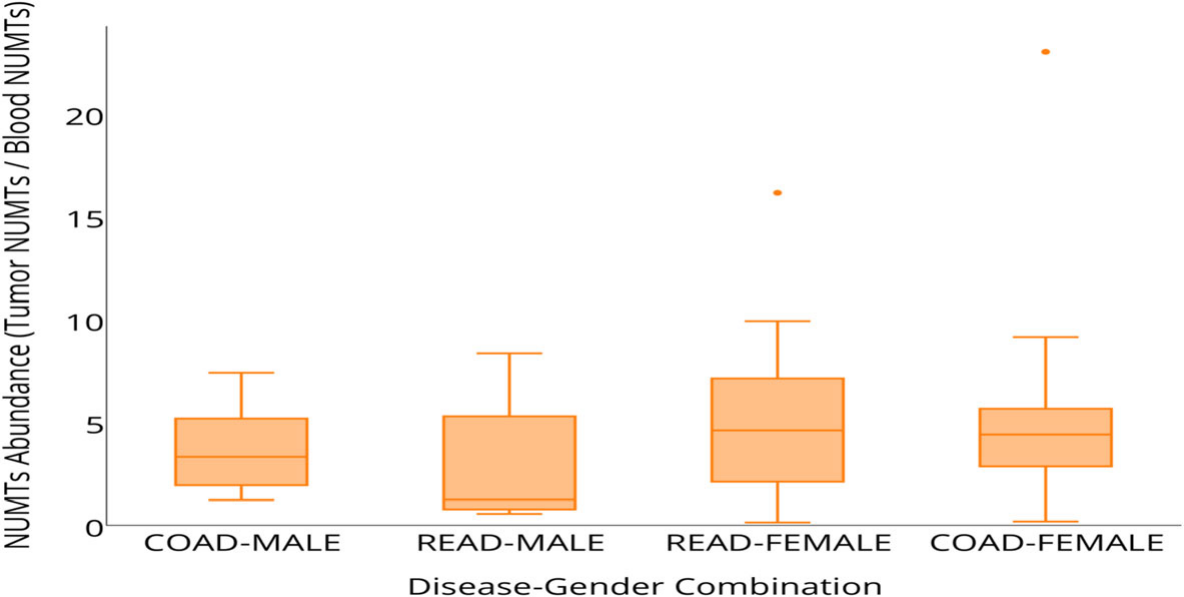
NUMT abundance across disease–sex combination. Colorectal tumors from women have higher NUMT abundance proportion (tumor NUMT/blood normal NUMT) than those from men. Women had a median NUMT fold change abundance of 4.52 (range 0.11 to 22.9) compared 3.1 for men (range 0.53 to 8.3). *COAD* colon adenocarcinoma, *READ* rectum adenocarcinoma. To investigate the sex difference in the NUMT abundances, an unequal variance *t*-test was performed on the raw NUMT proportions observed by the members of the two sex groups stratified by “blood-normal” and “primary tumor” classifications. The *P* value was 0.03 between males (*N* = 23) and females (*N* = 34) for blood-derived normal samples and 0.08 for tumor samples

Recent studies suggest hormonal regulation of mitochondrial functions [51]. Therefore, we delineated NUMT distribution among males and females. Colorectal tumors of women harbored more NUMTs than those of men (Fig. 4). Women had a median NUMT fold change proportion of 4.52 compared 3.1 for men. We conclude that tumors from women contained more NUMTs than those from men.

### NUMT abundance is associated with patient survival

**Fig. 5:**
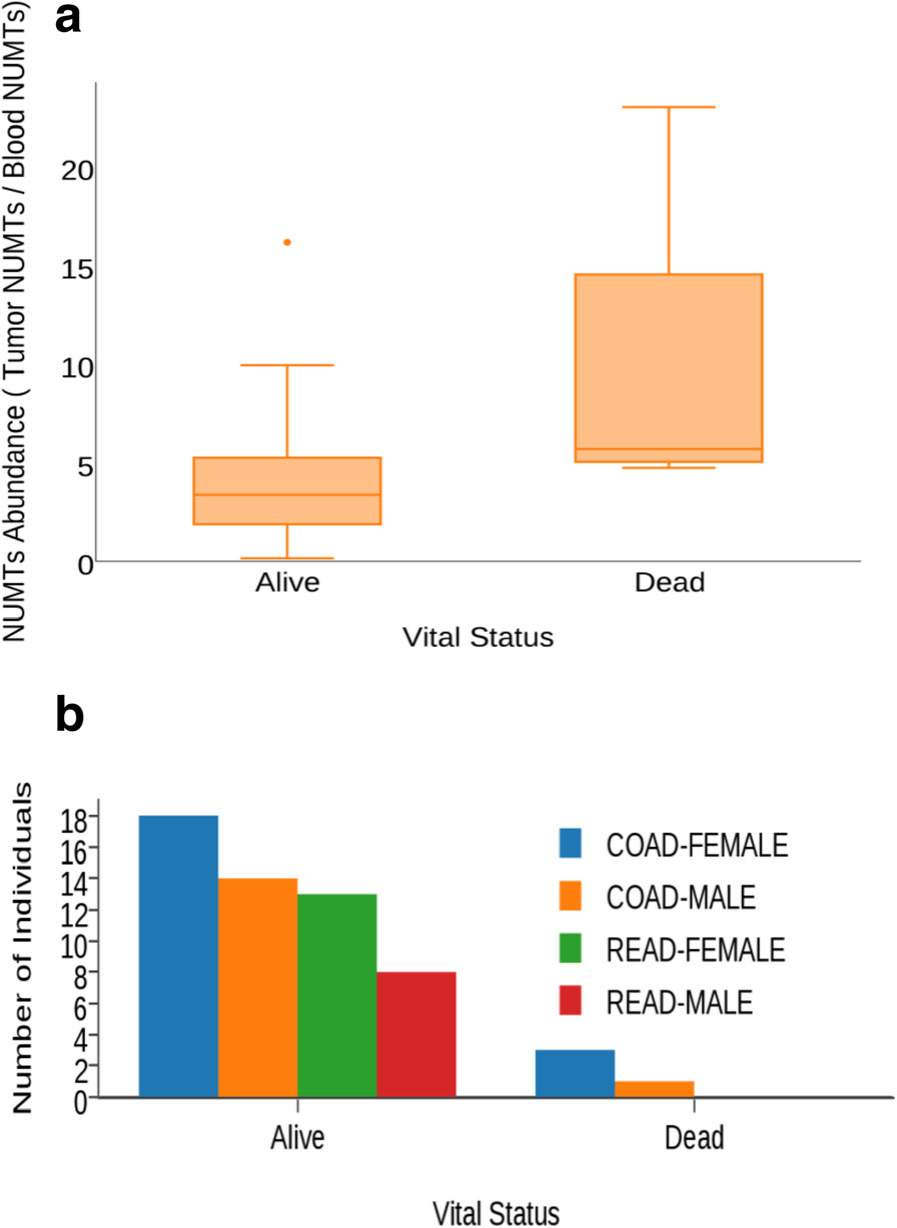
NUMT abundance distribution according to vital status. **a** Increased NUMT abundance in deceased individuals with colorectal tumors. Although a Mann–Whitney U test showed a *P* value of 0.04 between the NUMT abundances of the *Alive* and *Deceased* groups, the very small sample size (*N* = 4) of the deceased group warrants further investigation with datasets enriched for deceased vital status. **b** NUMT proportions categorized based on disease (colon and rectal) and sex combination. This vital status observation in combination with NUMT abundance among sex-specific samples appears to be an early indicator of death events among colorectal cancer women with higher proportions of NUMTs

**Fig. 6:**
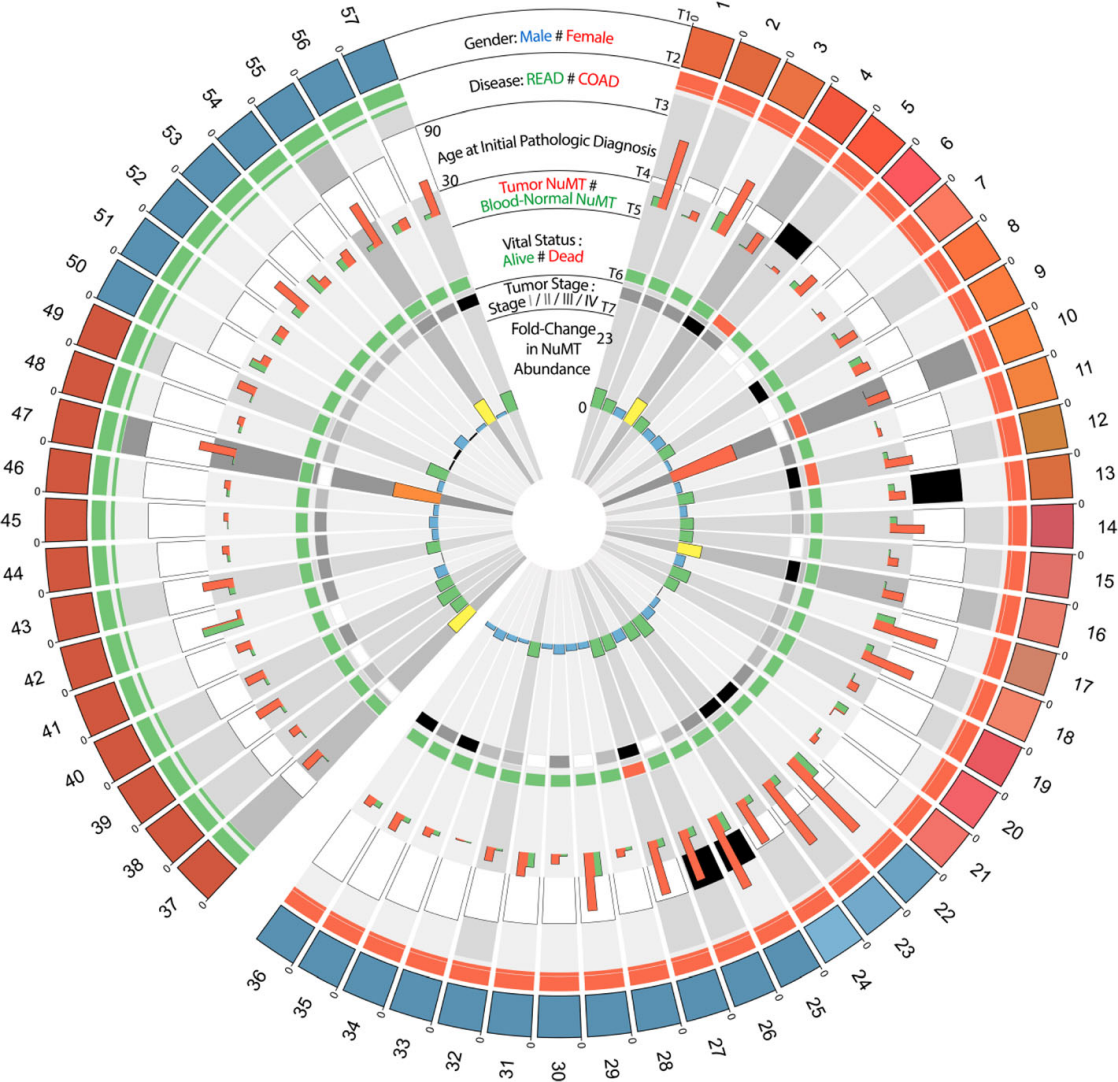
NUMT density in tumor and normal genomes (sorted by disease (T2), sex (T1), and age at initial pathologic diagnosis (T3)). Each peripheral node represents a TCGA sample whose blood-derived normal and tumor genomes were used in this study. From the outside to inside, tracks are ordered from 1 to 7 (T1–T7). *T1*: Sample sex where *red nodes* represent female and *blue nodes* represent male. *T2*: Disease type information. Rectal adenocarcinoma (*READ*) is rendered as *green bands* and colon adenocarcinoma (*COAD*) as *red bands. T3*: Age at initial pathologic diagnosis ranging from 30 to 90 years. *White* and *black filled bars* represent white and black race, respectively. *T4*: *Red columns* represent NUMT proportion in tumor genomes and *green columns* represent blood-derived normal NUMT proportion. *T5*: Vital status of the patients where *red* indicates deceased individuals and *green* alive status. *T6*: Stage of tumor represented in grey-scale—stage I *white*, stage II *grey*, stage III *dark grey*, and stage IV *black. T7*: Fold-change in NUMT abundance. Samples at <1-fold are rendered as *colored bands*; 1–4-fold, *blue*; 4–8-fold, *green*; 8–12-fold, *yellow*; 12–20-fold, orange; and >20-fold, *red*

It is conceivable that increased NUMT insertions may confer metastatic disease resulting in the death of patients. Therefore, we determined NUMT abundance and correlated it with patient survival. Figure 5 demonstrates that the NUMT abundance is amplified many fold in deceased individuals. Based on the survival rate among both sexes and vital status, there appears to be corroborating evidence that NUMTs in colorectal tumor genomes of women have a tendency to be amplified and are associated with patient survival. However, further analyses with larger sample sizes are required to make a definite conclusion. When overlaying these findings with circos integrators (Fig. 6; Additional file 2: Figures S1; Additional file 3: Figure S2), three of the four deceased individuals were reported to have pathologic tumor stage IIIC and IVA. Overall, except for one individual (women, stage I, deceased status), these results suggest that NUMT abundance increases with tumor grade and is significantly amplified in women.

### NUMT abundance positively correlates with GC content and gene density

**Fig. 7:**
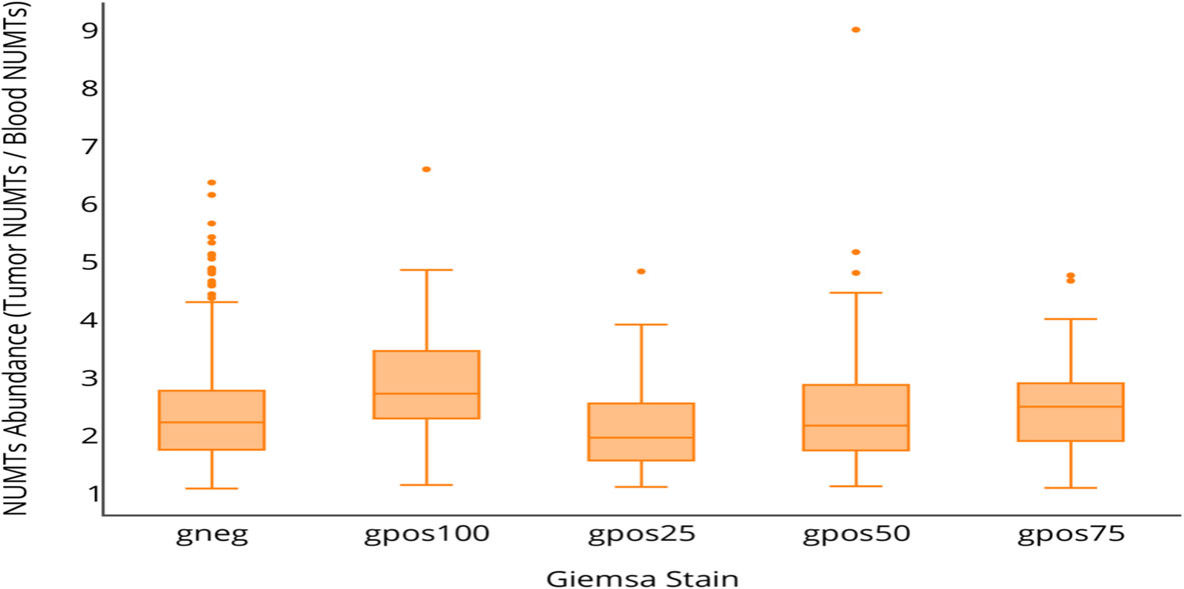
Correlation of NUMT abundance and GC content. Positive correlation between gene density and NUMT abundance. GC content is positively correlated with gene density; hence, regions of high GC content have higher relative gene density than regions of low GC content. Sample sizes (number of chromosome bands annotated by a certain Giemsa stain value) for the various Giemsa stain groups are as follows; *gneg*, *N* = 366; *gpos100*, *N* = 75; *gpos25*, *N* = 73; *gpos50*, *N* = 109; and *gpos75*, *N* = 82

GC content is positively correlated with gene density; hence, regions of high GC content have higher relative gene density compared to regions of low GC content [52]. Also, as Giemsa-negative chromosomal bands are gene-rich regions of DNA compared to bands positive for Giemsa staining [53], we extended our NUMT analyses to the chromosomal cytobands. Our study demonstrates a positive relationship between NUMT abundance and GC content of gene-rich regions, having an abundance fold-change of 4.2 or more (Fig. 7).

Overall, among the groups that had a fold change of 2 or more in NUMT abundance, although the low GC-content cytobands gpos50 and gpos100 were at the top of the NUMT abundance list for chromosomal cytoband windows, GC-rich “gneg” regions were prominent among cytobands exhibiting more than a 4.2-fold change in NUMT abundance. This pattern observed for the outlier data above 4.2 fold-change of NUMT abundance within GC-rich regions of gneg clearly shows a strong correlation between elevated NUMT abundance and GC content. Based on Fig. 7, as indicated by data points above the third quartile level, gneg regions are accountable for the greater than normal NUMT abundance, indicating a positive correlation between gene density and NUMT abundance. A previous study has shown that gpos100 sub-bands are also enriched with LINEs [54], and other researchers have demonstrated secondary amplification via LINE transposition to be an important mechanism of NUMT accumulation [11, 20]. Our results corroborate the empirical evidence. Clearly, a great deal of new research is needed to elucidate the causes and consequences of NUMT transposition. We hope that our discovery will serve as an impetus for such inquiry.

### Mitochondrial fragile sites associated with NUMTs

**Fig. 8:**
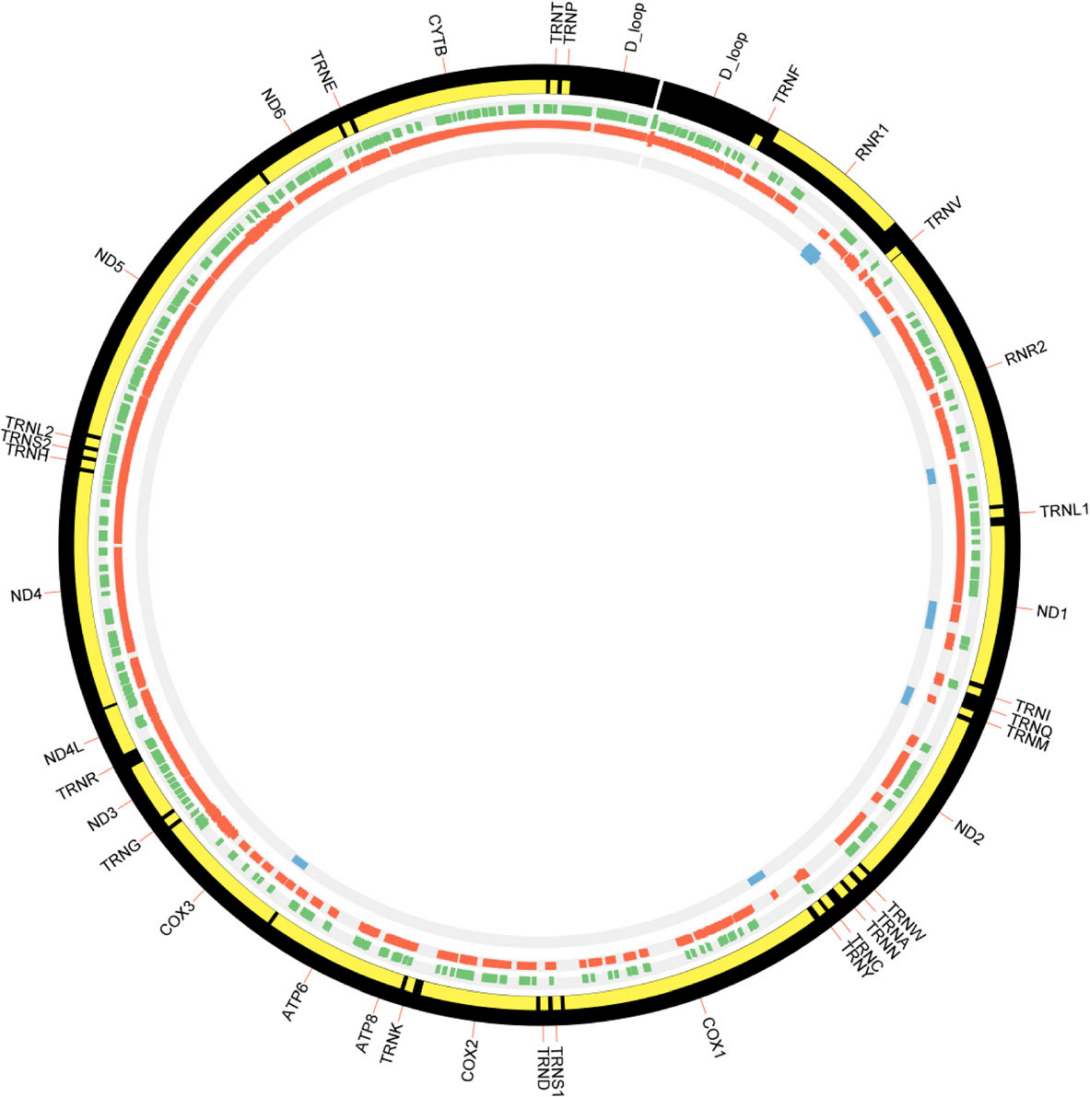
Top fragment sites of the mitochondrial genome identified in the nuclear genome. Genes on the mitochondrial genome and their potential fragile sites involved in the process of numtogenesis. Bands from the outside to inside represent: mitochondrial gene names; gene segments; numtogenesis regions of blood-derived normal samples (*green bands*); numtogenesis regions of primary tumor samples (*red bands*); numtogenesis regions unique to tumor samples but not observed in blood-derived normal samples (*blue bands*)

To identify fragile sites in the mitochondrial genomes that act as hotspots for mtDNA to immigrate into the nuclear genome, paired-end reads with one end mapping to the mitochondrial genome and the other end to the nuclear genome, NUMTs were further investigated. As illustrated in Fig. 8, fragile sites were identified within complex I (ND1) and complex IV (MT-CO1/COX1, COX III) mitochondrial regions, which are known for harboring mutations in different cancer sites. Based on the migration pattern of NUMTs, breakpoint sites in *COX1* and *ND1* are responsible for numtogenesis in the nuclear genome of colorectal cancer.

### *YME1L1* inactivation leads to increased numtogenesis

In the yeast *Saccharomyces cerevisiae*, *YME1* is reported to be an important suppressor of mtDNA migration to the nucleus [55]. Interestingly, the *YME1L1* gene encodes the human homologue of yeast mitochondrial AAA (ATPases associated with diverse cellular activities) metalloprotease, Yme1p. YME1L is a functional homologue of Yme1p, with conserved roles in mitochondrial assembly, integrity, and DNA metabolism [56]; however, its function in suppressing NUMTs is not known.

**Fig. 9:**
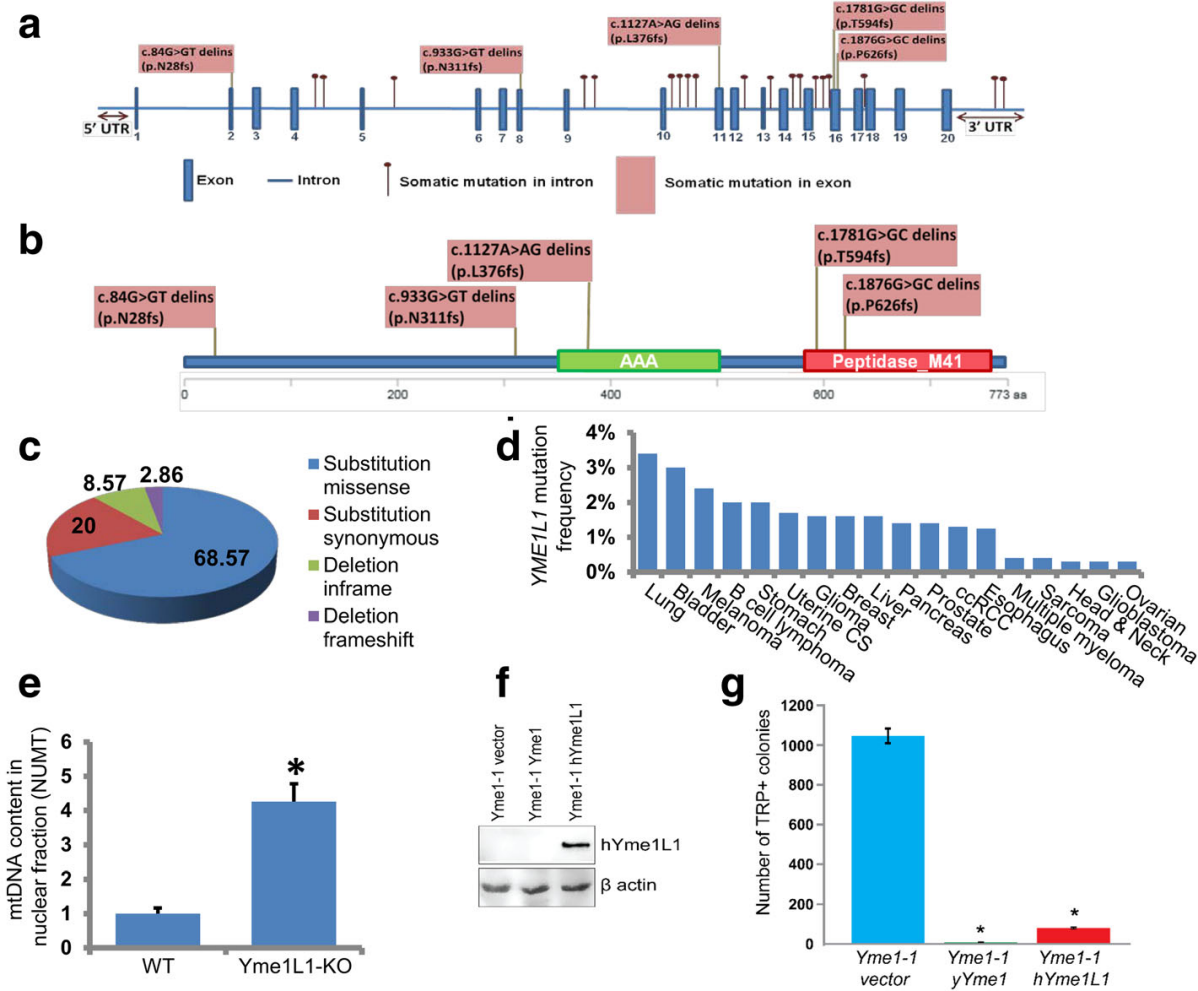
Human *YME1L1* inactivation leads to increased numtogenesis. TCGA tumor sample data used in this study were screened for mutations in the *YME1L1* gene. A total of 24 mutations (five exonic, 17 intronic, and two in the 3′ UTR) in *YME1L1* were identified. All five exonic mutations were frameshift mutations. **a**, **b** The position of these mutations in the *YME1L1* gene (**a**) and protein (**b**). **c** The total colorectal cancer samples available in TCGA database were analyzed on 4 April 2016 for mutations in Yme1L1 and their types determined. **d** Mutations in Yme1L1 in other cancers were also analyzed using the cBioPortal database. Altered frequency of Yme1L1 mutations in different cancer types are represented. **e** mtDNA content in nuclear fractions, i.e., NUMT accumulation was analyzed in wild-type (*WT*) and YME1L1 knockout (*Yme1L1*-*KO*) human cell lines. NUMT accumulation was about fourfold increased in YME1L1-KO cells compared with wild-type cells. Data are expressed as mean ± standard error of the mean (sem); *P < 0.05, Student’s *t*-test. **f**, **g** The yeast *PTY33-Yme1-1* (*ρ+, TRP1*) strain was transformed with empty plasmid and plasmids expressing yYme1 and hYme1L1 with *URA* marker as indicated. Transformed cell colonies were selected by synthetic dropout medium lacking URA. **f** Whole cell lysate from the *Yme1-1* vector, *Yme1-1* yYme1, and *Yme1-1* hYme1L1 strains was subjected to SDS-PAGE and western blotting was performed with antibodies against hYme1L1 and β-actin. **g** *Yme1-1* vector, *Yme1-1* yYme1, and *Yme1-1* hYme1L1 cells were spread on plates lacking tryptophan; the experiment was performed three times in triplicate. Data are expressed as mean ± sem; *P < 0.05, Student’s *t*-test

We conducted in silico *YME1L1* mutation analysis in all colorectal cancer cases (n = 57) used for NUMT analysis in this study. We determined that of 57 CRC tumors, ~16% contained mutations in *YME1L1*. A total of 24 mutations (five exonic, 17 intronic, and two in the 3′ UTR) in *YME1L1* were identified. All five exonic mutations were frameshift mutations (Fig. 9a, b).

We expanded our analysis of Yme1L1 in TCGA database. This analysis revealed a high incidence of Yme1L1 mutations in CRC (Fig. 9c). The mutations in Yme1L1 include missense and synonymous substitutions and inframe and frameshift deletions (Fig. 9c). Most of the mutations in Yme1L1 in human colorectal cancer fall into two categories: missense substitutions (~68%) and synonymous substitutions (20%). The relative distribution of various mutations is summarized as a pie chart in Fig. 9c. We also analyzed Yme1L1 mutations in other human cancer types and observed a high mutation frequency in all the tested cancer types (Fig. 9d).

We determined whether inactivation of the human *YME1L1* gene increases NUMT formation. For this, we created a *YME1L1* knockout in a human cell line using *YME1L1* gene-specific CRISPRs. We prepared nuclear fractions free of mitochondrial contamination and quantified the amount of mtDNA present in the nuclear fraction of these cells. We observed a strikingly increased amount of mtDNA in the nuclear fraction of *YME1L1* knockout cells compared to wild-type cells (Fig. 9e). These results identify *YME1L1* as the first NUMT suppressor gene in humans and suggest that inactivation of *YME1L1* leads to increased numtogenesis.

### Human homologue of *YME1* suppresses migration of mtDNA to the nucleus

We asked whether the phylogenetically conserved role of human *YME1L1* can rescue the migration of mtDNA in a yeast strain in which *YME1* is disrupted. We utilized the *Yme1-1* yeast strain, which harbors a mutation which leads to inactivation of the *YME1* gene [57]. In this strain, the auxtotrophic endogenous nuclear *TRP1* gene is deleted and inserted into the mitochondrial genome. Since the required transcription machinery for *TRP1* is only present in the nucleus, the mitochondrially inserted *TRP1* gene is only functional when it migrates to the nucleus, permitting analysis of mtDNA migration to the nucleus [57, 58].

To determine whether human Yme1L1 is expressed in the *Yme1-1* strain, western blotting was performed. Indeed, hYme1L1 was expressed in the *Yme1-1* hYme1L1 strain (Fig. 9f). When the *Yme1-1* vector was plated under tryptophan selection, it showed a significantly large number of colonies (Fig. 9g). These data support a previous observation that migration of mtDNA to the nucleus is high in *Yme1-1* cells [57]. *Yme1-1* cells expressing yeast Yme1 (yYme1) display only a few (<50) tryptophan-positive colonies, suggesting that accumulation of mtDNA fragments in the nucleus in the *Yme1-1* strain was prevented to a greater degree by re-introducing yYme1 (Fig. 9g). The same number of *Yme1-1* cells harboring hYme1L1 also produced significantly low number of tryptophan-positive colonies compared to *Yme1-1* cells (Fig. 9g). However, the number of typtophan-positive colonies in this case was a little higher (>100) compared to yYme1-expressing cells. This suggests that hYme1L1 can partially rescue the phenotype of mtDNA escape in *Yme1-1*. We conclude that hYme1L1 suppresses migration of mtDNA to the nucleus.

## Discussion

Numtogenesis, a natural phenomenon leading to migration of mitochondria, mitochondrial proteins, mtRNA, or mtDNA into the nucleus, is an ongoing cellular process reported in eukaryotic cells [2–9]. Although the occurrence of NUMT and the phenomenon of numtogenesis have been reported, its role in cellular and organismal function and in human health and disease remains relatively unexplored. Our study revealed that somatic NUMTs are frequently found in colorectal cancer. Consistent with our finding, two previous studies have associated NUMTs with carcinogenesis; one that found NUMTs containing LINEs in rat and mouse tumors [20] and a study of a cervical carcinoma cell line [19].

We provide evidence for increased NUMT insertions in the nuclear genomes of colorectal adenocarcinomas relative to matched control samples. NUMT occurrence was influenced by pathological cancer stage and sex between tumor and control groups. Germ line NUMT insertions leading to diseases have been identified [59]. These diseases include severe plasma factor VII deficiency and bleeding diathesis [60], mucolipodosis IV [61], Usher syndrome [62], and a rare Pallister-Hall syndrome [63]. These NUMT insertions were found in coding genes. NUMT insertions in these genes can reduce cellular fitness, leading to cellular dysfunction-induced cell death, which may underlie these human diseases [64]. Conceivably, somatic NUMT insertion in tumor suppressor gene(s) may disrupt pathways which can contribute to tumorigenesis. Similarly, NUMT may activate oncogene(s) involved in tumor development. Indeed, integration of mtDNA fragments in the *MYC* locus in HeLa cells [65] and in the nuclear genome of mouse embryonic fibroblasts [66] has been identified. It appears that the integration of mtDNA in the nuclear genome of mouse embryonic fibroblasts led to the malignant transformation [66].

Increased somatic NUMT insertion in the nuclear genome of tumors may be associated with mitochondrial dysfunction. Mitochondrial dysfunction is a consistent feature of a variety of tumors and is described to be a hallmark of cancer [67–72]. We have previously demonstrated that mitochondrial dysfunction induces genomic instability in the nucleus [30, 31, 33]. However, genetic instability associated with mitochondrial dysfunction has been described to be point mutations or chromosomal aneuploidy [73–75]. The nuclear genome instability was induced due to increased oxidative stress caused by the changes in the nucleotide pool [30]. Hadler et al. [76] proposed “a unitary hypothesis for carcinogenesis”, speculating that a breakdown of mito-nuclear symbiosis leads to development of cancer; they suggested release of mtDNA due to damage to the mitochondrial membranes. Using a sliding window approach, we determined NUMT insertion in tumor genomes. When we searched for the origin of these nuclear NUMT landing sites among colorectal cancer samples, we found three potential fragile sites within the mitochondrial genes *ND1*, *COX1*, and *COX3*, whose association with colorectal cancer has already been well investigated [77, 78]. Interestingly, in most females NUMTs originated from the same mitochondrial regions while in males NUMTs originated from different regions of mtDNA.

**Fig. 10:**
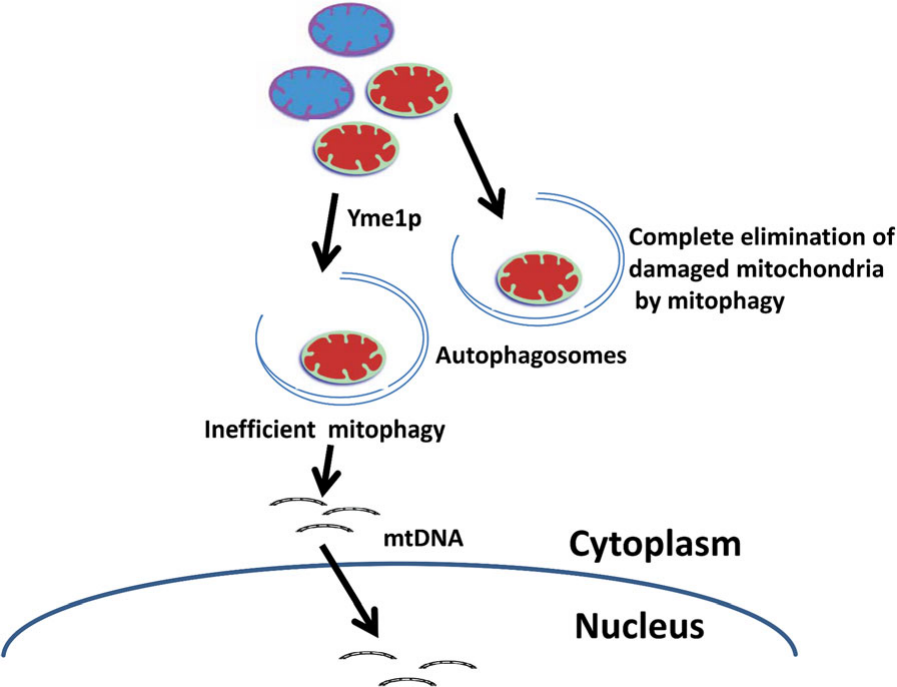
Mechanism underlying numtogenesis. Our observations support the role of Yme1 in numtogenesis. The role of Yme1 in the regulation of mitophagy is well established. Mitophagy is a stringent mechanism that controls the quality of mitochondria in cells by degrading dysfunctional mitochondria. Compromised mitophagy due to altered Yme1 function leads to accumulation of undigested mtDNA in the cytoplasm that ultimately ends up in the nucleus, a process we have named numtogenesis

In the yeast *S. cerevisiae*, mitochondrial dysfunction leads to increase escape of mtDNA into the nucleus [57]. Mutations in the *YME1* gene induce migration of mtDNA to the nucleus [57]. Yme1p is a multifunctional protein, controlling mitochondrial quality, which plays an important role in mitochondrial biology, including assembly of mitochondrial respiratory complexes, and importantly in mitophagy [79, 80]. We identified that 16% of analyzed colorectal tumors contained mutations in the *YME1L1* gene (Fig. 9a, b). Mutations in Yme1L1 were also found in a variety of other types of tumors (Fig. 9d). We demonstrate that the human homolog of yeast Yme1 functions as a “NUMT suppressor”. Human YME1L1, when expressed in a mutant Yme1 yeast strain, reduces the migration of mtDNA to the nucleus (Fig. 9g). These observations support *YME1LI* as a NUMT suppressor gene in humans whose inactivation leads to increased numtogenesis. Yme1 removes the damaged or dysfunctional mitochondria by mitophagy and maintains a healthy pool of mitochondria in the cell [81]. These observations implicate mitophagy in mediating accumulation and transfer of mtDNA into the nuclear genome. These observations lead us to suggest that the mechanism underlying numtogenesis may involve Yme1-mediated mitophagy. Mitophagy is a stringent mechanism that controls the quality of mitochondria in cells [81]. Compromised mitophagy due to loss of Yme1 [80] or an acid endonuclease DNase IIα [82] can lead to accumulation of incompletely digested mtDNA in the cytoplasm that ultimately ends up in the nucleus (Fig. 10).

It is conceivable that a direct physical association or fusion between the mitochondrial and nuclear membranes and encapsulation of mitochondria in the nucleus [83, 84] may contribute to numtogenesis. Observations supporting encapsulation of mitochondria in the nucleus have been reported [2–5, 7–9]. The nuclear envelope breaks down during mitosis, leading to disruption of the physical barrier seperating the nucleoplasm and cytoplasm [85]. This stage of the cell cycle can provide an opportunity for mitochondria to enter into the nucleus. Furthermore, cancer cells often exhibit a ruptured nuclear envelope [86]. Decreased expression of lamins, important constituents of the nuclear membrane, contributing to nuclear rupture in cancer cells, has also been reported [87]. Lamins in the nuclear membrane bind to chromatin and hold chromosomes in place and thus reduce chromosome breakage [88]. Indeed, patients with laminopathy resulting from reduced lamin expression contain mitochondria in the nucleus. It is likely that loss of lamin expression in cancer cells helps migration of mitochondria into the nucleus, resulting in eventual integration of mtDNA into the nuclear genome. This phenomenon might be a survival mechanism for cancer cells [13].

It is unclear how numtogenesis alters the nuclear genome functions. Tsuji and coauthors [25] hypothesized that the underrepresentation of d-loop NUMTs in the germline may be due to protein binding sites located in this region, which may interrupt mtDNA fragmentation and immigration to the nucleus. We propose an alternative mechanism which involves structural alteration of the chromosome. Under this model, the inserted mitochondrial d-loop insertion functions as a telomeric t-loop, whereby it stabilizes a double-strand break and truncates the chromosome arm, resulting in aneuploidy. This mechanism involves transfer of the mtDNA displacement loop (d-loop) to the nuclear genome, where it could promote aneusomy by functioning as a telomeric “t-loop” structure, which typically caps the linear DNA molecule with a triple-stranded loop. The insertion of a mitochondrial d-loop could therefore directly interfere with the secondary structure of open chromatin and histone binding sites, leading to genome instability and/or dysregulation of gene expression. The second putative mechanism involves a mismatch between nucleotide composition between the mtDNA origin and nuclear DNA insertion site, which again draws from comparisons with work of Tsuji and coauthors [25]. Previous work has shown that non-homologous recombination and transposable element insertion can lead to genome instability by modifying local methylation patterns and altering molecular thermodynamics, which could lead to dysregulation of the cell cycle and aneuploidy. These two putative mechanisms are not mutually exclusive and we anticipate that novel mechanisms will be revealed through further investigation.

## Conclusions

Our study reveals that numtogenesis plays an important role in the development of cancer and that NUMTs may serve as a biomarker for tumorigenesis. This study also identifies *YME1L1* as the first NUMT suppressor gene in human and demonstrate that inactivation of *YME1L1* induces migration of mtDNA to the nuclear genome. Exploration of mtDNA migration into the cancer genome should provide impetus for further studies to identify the mechanism(s) underlying numtogenesis.

## Additional files

Additional file 1: Table S1. NUMT abundance in all the colorectal tumor samples (n = 57) used in this study. (XLSX 14 kb)

Additional file 2: Figure S1. NUMT density in tumor and normal genomes (sorted by disease (T2) and fold change in NUMT abundance (T7)). Each peripheral node represents a TCGA sample whose blood-derived normal and tumor genomes were used in this study. From the outside to the inside, tracks are ordered from 1 to 7 (T1–T7). *T1*: Sample gender where *red nodes* represent female and *blue nodes* represent male. *T2*: Disease type information. Rectal adenocarcinoma (*READ*) is rendered as *green bands* and colon adenocarcinoma (*COAD*) as *red bands. T3*: Age at initial pathologic diagnosis ranging from 30 to 90 years. *White* and *black filled bars* represent white and black race of the individual, respectively. *T4*: *Red columns* represent NUMT proportion in tumor genomes and *green columns* represent blood-derived normal NUMT proportion. *T5*: Vital status of the patients—*red* for deceased individuals and *green* for alive status. *T6*: Stage of tumor represented in grey scale—stage I *white*, stage II *grey*, stage III *dark grey*, stage IV *black. T7*: Fold change in NUMT abundance. Samples with <1-fold are rendered as *colored bands*: 1–4-fold, *blue*; 4–8-fold, *green*; 8–12-fold, *yellow*; 12–20-fold, *orange*; >20-fold, *red*. (TIF 10991 kb)

Additional file 3: Figure S2. NUMT density in tumor and normal genomes (sorted by disease (T2) and pathologic tumor stage (T6)). Each peripheral node represents a TCGA sample whose blood-derived normal and tumor genomes were used in this study. From the outside to the inside, tracks are ordered from 1 to 7 (T1–T7). *T1*: Sample gender where *red nodes* represent female and *blue nodes* represent male. *T2*: Disease type information. Rectal adenocarcinoma (*READ*) is rendered as *green bands* and colon adenocarcinoma (*COAD*) as *red bands. T3*: Age at initial pathologic diagnosis ranging from 30 to 90 years. *White* and *black filled bars* represent white and black race of the individual, respectively. *T4*: *Red columns* represent NUMT proportion in tumor genomes and *green columns* represent blood-derived normal NUMT proportion. *T5*: Vital status of the patients—*red* for deceased individuals and *green* for alive status. *T6*: Stage of tumor represented in grey scale—stage I *white*, stage II *grey*, stage III *dark grey*, stage IV *black. T7*: Fold change in NUMT abundance. Samples with <1-fold are rendered as *colored bands*: 1–4-fold, *blue*; 4–8-fold, *green*; 8–12-fold, *yellow*; 12–20-fold, *orange*; >20-fold, *red*. (TIF 10662 kb)

## Abbreviations

BAM: Binary alignment
CGHub: Cancer Genomics Hub
COAD: Colon adenocarcinoma
CRC: colorectal cancer
mtDNA: Mitochondrial DNA
mtRNA: Mitochondrial RNA
NUMT: nuclear mtDNA sequence
QC: quality control
READ: Rectum adenocarcinoma
TCGA: The Cancer Genome Atlas
