# Supplementary figures and images for: Migration of mitochondrial DNA in the nuclear genome of colorectal adenocarcinoma
Source: Genome Med. 2017 Mar 29;9:31. doi: 10.1186/s13073-017-0420-6 (PMC5370490; doi:10.1186/s13073-017-0420-6)

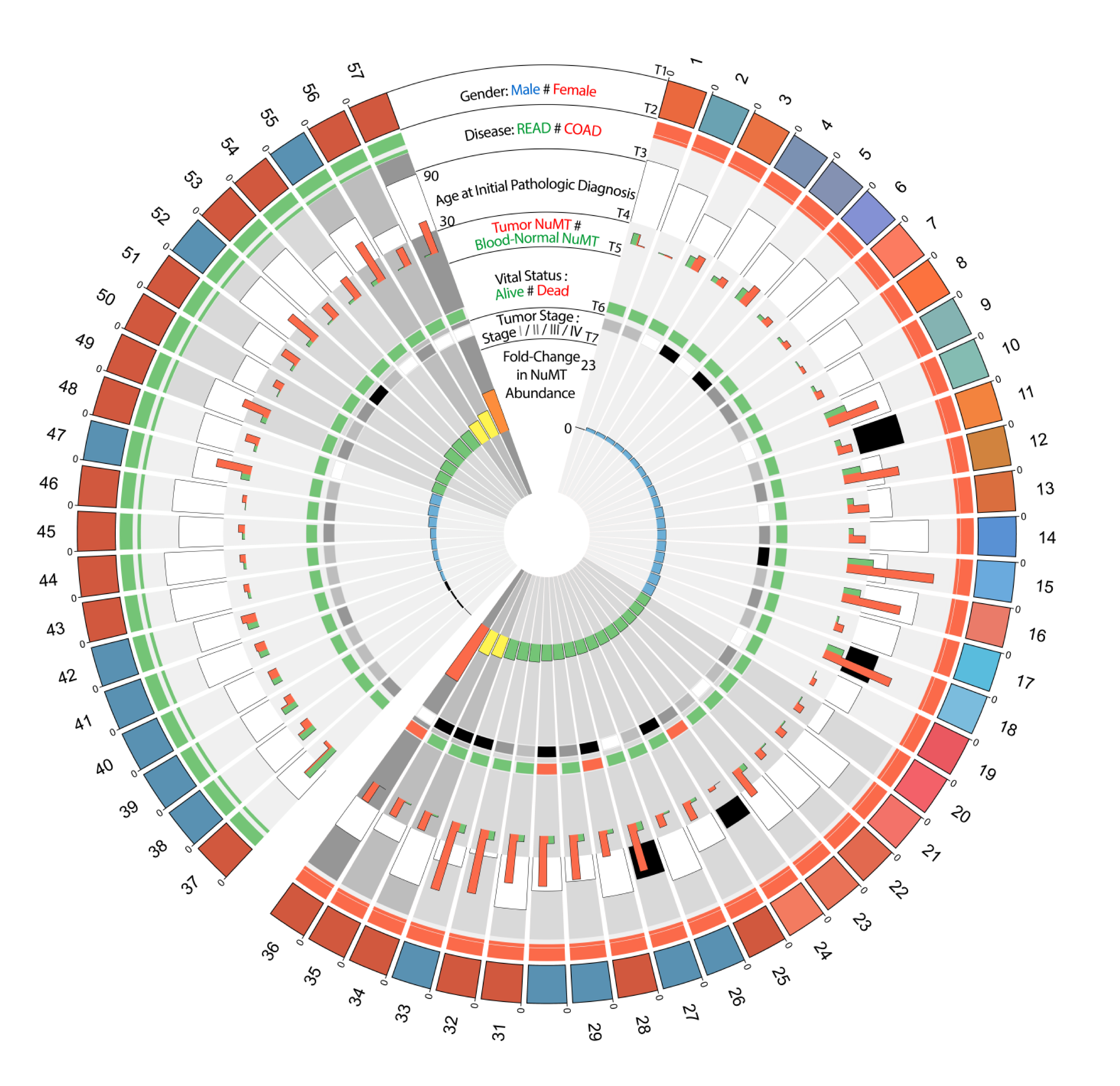

Supplement: Supplementary file 2 — NUMT density in tumor and normal genomes (sorted by disease (T2) and fold change in NUMT abundance (T7)). Each peripheral node represents a TCGA sample whose blood-derived normal and tumor genomes were used in this study. From the outside to the inside, tracks are ordered from 1 to 7 (T1–T7). T1: Sample gender where red nodes represent female and blue nodes represent male. T2: Disease type information. Rectal adenocarcinoma (READ) is rendered as green bands and colon adenocarcinoma (COAD) as red bands. T3: Age at initial pathologic diagnosis ranging from 30 to 90 years. White and black filled bars represent white and black race of the individual, respectively. T4: Red columns represent NUMT proportion in tumor genomes and green columns represent blood-derived normal NUMT proportion. T5: Vital status of the patients—red for deceased individuals and green for alive status. T6: Stage of tumor represented in grey scale—stage I white, stage II grey, stage III dark grey, stage IV black. T7: Fold change in NUMT abundance. Samples with <1-fold are rendered as colored bands: 1–4-fold, blue; 4–8-fold, green; 8–12-fold, yellow; 12–20-fold, orange; >20-fold, red. (TIF 10991 kb) [file 13073_2017_420_MOESM2_ESM.tif]

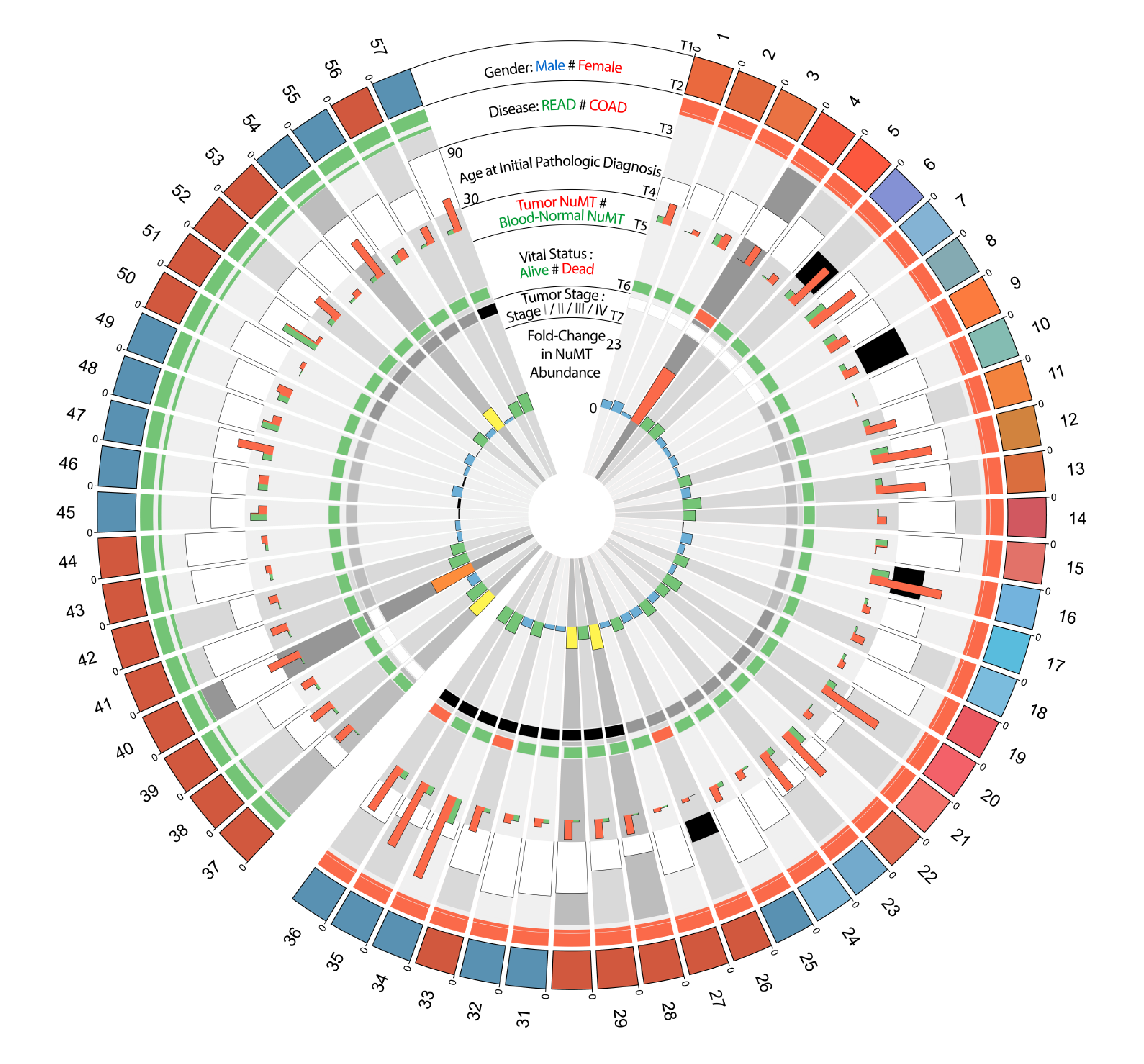

Supplement: Supplementary file 3 — NUMT density in tumor and normal genomes (sorted by disease (T2) and pathologic tumor stage (T6)). Each peripheral node represents a TCGA sample whose blood-derived normal and tumor genomes were used in this study. From the outside to the inside, tracks are ordered from 1 to 7 (T1–T7). T1: Sample gender where red nodes represent female and blue nodes represent male. T2: Disease type information. Rectal adenocarcinoma (READ) is rendered as green bands and colon adenocarcinoma (COAD) as red bands. T3: Age at initial pathologic diagnosis ranging from 30 to 90 years. White and black filled bars represent white and black race of the individual, respectively. T4: Red columns represent NUMT proportion in tumor genomes and green columns represent blood-derived normal NUMT proportion. T5: Vital status of the patients—red for deceased individuals and green for alive status. T6: Stage of tumor represented in grey scale—stage I white, stage II grey, stage III dark grey, stage IV black. T7: Fold change in NUMT abundance. Samples with <1-fold are rendered as colored bands: 1–4-fold, blue; 4–8-fold, green; 8–12-fold, yellow; 12–20-fold, orange; >20-fold, red. (TIF 10662 kb) [file 13073_2017_420_MOESM3_ESM.tif]
